# Beat People but Not Face: The Role of Perceived Face Threat in the Influence of Abusive Supervision on Employee Feedback Seeking

**DOI:** 10.3390/bs13060462

**Published:** 2023-06-02

**Authors:** Qin Chen, Shilong Liao, Long Lin, Li Zhang

**Affiliations:** 1 School of Economics and Management, Harbin Institute of Technology, Harbin 150006, China; 2 School of Economics and Management, Lanzhou Institute of Technology, Lanzhou 730050, China; 3 School of Economics and Management, Lanzhou University of Technology, Lanzhou 730050, China

**Keywords:** abusive supervision, feedback seeking behavior, perceived face threat, self-affirmation, self-handicapping

## Abstract

One of the purposes for superiors to abuse subordinates is to obtain a positive response from subordinates by conveying a negative attitude. However, abusive behavior cannot guarantee positive behaviors due to the differences in subordinates’ characteristics, such as feedback seeking. Based on the conservation of resources (COR) theory, this study explores the relationship between abusive supervision by superiors and feedback seeking by subordinates in East Asian cultures. Questionnaires were collected from multiple time points and multiple sources. Datum analysis was performed on 318 paired questionnaires between employees and direct supervisors. The results showed that: (1) Employees’ perceived face threat has a mediating effect on the relationship between abusive supervision and feedback seeking. (2) Self-affirmation of subordinates positively moderates the relationship between abusive supervision and perceived face threat. (3) Self-handicapping of subordinates positively moderates the relationship between perceived face threat and feedback seeking. This not only explains the mechanism of perceived face threat in the influence of abusive supervision on employees’ feedback-seeking behavior, but also reveals the boundary effect of employees’ self-affirmation and self-handicapping characteristics in it, which expands the theoretical explanation framework of the influence of abusive supervision on employees’ feedback-seeking behavior and also provides new ideas for managers to better implement management in the organization.

## 1. Introduction

There is an old Chinese saying: “To hit people not to hit their faces, to curse at people does not expose their shortcomings”, which emphasizes the importance of the face in the hearts of the Chinese [1]. However, as a typical representative of the “dark side” of leadership behavior, abusive supervision was prevalent in organizations, especially in East Asian organizations subject to face-oriented cultures such as “absolute monarchy” and “centralization”, which violated the organizational ethics and posed a threat to the face of victims [2,3]. Recently, in a study taking Asian individuals as a sample, it was found that face threa t sensitivity played a positive role in regulating the shame of employees caused by abusive supervision, and further affected the positive effect of abusive supervision on employee performance improvement [1]. Peer abusive supervision also positively affects the third party’s impression management of employees through the third party’s face threat [4]. These studies show that although abusive supervision has posed a certain threat to the face of victims, it has strengthened the response of the victims’ positive behavior. Meanwhile, face threat has played a positive role in employees’ responses caused by abusive supervision. According to Goffman’s research, the face selection strategy related to losing face focuses on how to maintain, preserve or avoid losing face, which includes such withdrawal behaviors as acting according to rules, being cautious, limiting your actions, and not making promises easily, not boasting or even not taking action [5]. Previous studies have shown that abusive supervision by the boss will lead to employees’ psychological experience of depression, tension, decreased job satisfaction and happiness, as well as decreased organizational commitment [6]. At the same time, it will also cause a series of negative behavioral reactions, such as retaliating against the boss or hurting the organization and other deviant behaviors, defensive silence, feedback avoidance behavior, etc. [7]. This is detrimental to employees’ personal development and affects enterprises’ sustainable development [8]. According to the approach-avoidance perspective, after abusive treatment by superiors, employees’ deviant behaviors that retaliate against superiors or hurt the organization are called avoidance-oriented negative responses. In contrast, defensive silence and feedback avoidance are called avoidance-oriented positive responses [9]. Most previous studies have explored the mechanism of employees’ approach-oriented behavioral responses to abusive supervision, such as employees’ attribution style [10], psychological distress [11], self-control ability [6], etc. However, these studies paid little attention to the mechanism of employees’ avoidance-oriented behavioral responses. In fact, the superiors in the organization often control the power resources such as employee promotion and continued employment. Therefore, employees who have suffered abusive treatment from their superiors may be afraid of further retaliation from their superiors and are more inclined to make evasive behavioral responses [12]. This suggests that the threat of losing face caused by abusive supervision may lead to increased negative behaviors and decreased positive behaviors, which may be contrary to previous research conclusions. Thus, it is necessary to further explore the role and mechanism of perceived face threat in the effects of abusive supervision on employee behavior.

Abusive supervision is essentially a kind of negative feedback behavior to correct errors [12], and some leaders, pro-organization, hope to urge employees with poor management performance to improve their performance by abusing them [13]. Therefore, whether the victims can understand their problems and improve themselves after abusive supervision has become the key to affecting the effectiveness of abusive supervision. According to the research of Ashford et al., (1983), employees can actively seek the information they need in their work from their superiors, that is, feedback-seeking behavior [14]. It has a positive effect on both individuals and organizations, such as promoting the socialization of new employees, establishing high-quality leadership member exchange relationships, promoting career development, improving management effectiveness and achieving innovative performance [15]. Therefore, how to motivate employees to seek feedback actively has been the focus of academic attention. However, because seeking feedback is at risk of damaging self-image and self-esteem and requires extra effort and reasoning costs, individuals’ motivation and willingness to seek feedback are greatly weakened [16]. It has been pointed out that abusive supervision will lead to the reduction of feedback-seeking behavior of employees, but the mechanism of this is rarely clarified. Only Shen et al., (2020) pointed out through research that abusive supervision can reduce employees’ feedback-seeking behavior by affecting their organizational self-esteem [17]. Although self-esteem and face are related to personal self-worth [7,18,19], the two cannot be confused. Self-esteem is an individual’s affirmation and conviction of self-worth. It is a subconscious and automatic self-evaluation formed by accumulating much experience. Therefore, it is relatively stable and requires less recognition from others [20]. The face is an intrinsic social self-worth that needs to be confirmed by others and emphasizes social interaction and situational [5]. Although influential individuals will affect employees’ organization-based self-esteem, it can be seen from previous studies that this impact may require a large amount of organization-related experience to achieve. Therefore, in Chinese culture, the concept of face is more important than that of other countries and regions, especially in Western countries [5]. The introduction of the face into the research framework of the damage to employees’ self-worth caused by abusive supervision is a theoretical perspective that is beneficial to better understand how abusive supervision affects employees’ feedback-seeking behavior through face threat.

In addition, Tepper, the proponent of abusive supervision, pointed out that because abusive supervision results from the subjective perception of subordinates, the individual difference in abusive supervision perception is an important content worth studying [21]. For example, in the face of the same management behavior from the same superior, why different subordinates may perceive the differentiated level of abusive supervision [21]. This suggests that status and trait differences of employees may be the key to how they perceive superior management behavior. Based on this, this study further expands the research on the perceived differences in abusive supervision from the two dimensions of individual status and characteristics.

Through abusive behavior, superiors can convey information about inadequate ability and low performance to subordinates, which will threaten the latter’s image and status. Therefore, self-affirmation has been widely concerned because it can help individuals cope with threats [22]. This means that when an individual encounters a threat, he/she maintains self-integrity by affirming his/her self-worth in fields unrelated to the threat; that is, he/she believes that he/she is good on the whole: morally noble and socially adapted [23]. However, when this “good person” image is threatened, his response is often to restore his self-worth to maintain his integrity, that is, to make up for the defects of his B-side with the advantages of his A-side so as to rebalance the self-system. Furthermore, individuals with high self-affirmation respond positively to threatening information and adverse situations by “learning from each other” to prevent defensive reactions that may be detrimental to their development. Therefore, from the perspective of resource conservation, this study introduced the individual state variable of self-affirmation to explore the moderating effect of self-affirmation when abusive supervision by superiors threatens subordinates’ face resources, thus enriching the boundary conditions of the influence of abusive supervision on perceived face threat.

In addition, failure may imply self-worth for individuals. For example, low ability is often considered the cause of failure, and low ability is equivalent to low self-worth. Therefore, when self-worth is threatened, subordinates may try to avoid failure or even give up the opportunity to pursue success in order to protect their sense of self-worth [24]. Thus, when faced with achievement situations, individuals may be motivated to protect their self-worth and take the self-handicapping strategy. As self-handicapping is a kind of individual trait, people with high self-handicapping prefer self-protection [25,26]. Therefore, when individuals with high self-handicapping are faced with face threats due to abusive supervision by their superiors, the self-handicapping strategy will be activated for self-worth protection. Therefore, this study introduced the trait of self-handicapping into the research of abusive supervision and explored the moderating effect of self-handicapping on the influence of perceived face threat on feedback seeking.

In conclusion, this paper makes the following contributions: (1) For the unique face culture of East Asian countries, based on the face theory, this paper explores the mediating role of perceived face threat between abusive supervision and subordinates’ feedback seeking. It expands the mechanism of the influence of abusive supervision on feedback-seeking behavior. (2) Based on self-worth theory and conservation of resources (COR) theory, the boundary effect of self-affirmation on abusive supervision and perceived face threat was discussed from the perspective of subordinates’ status. (3) According to the COR theory, from the perspective of subordinates’ personality trait, the moderating effect of self-handicapping on perceived face threat and feedback seeking is discussed.

## 2. Theoretical Background and Research Hypothesis

### 2.1. Abusive Supervision and Perceived Face Threat

Abusive supervision refers to persistent verbal and non-verbal hostile behavior (but not physical contact) perceived by subordinates, such as ridicule, public criticism, belittling and questioning of subordinates’ competence [27]. Relevant studies have pointed out that abusive supervision, as a stressor, can stimulate different coping behaviors in individuals [21]. Face is a social psychological construction rooted in culture. It refers to the social dignity or public image claimed by an individual and recognized by others. It is an important factor affecting the psychology and behavior of East Asians [28]. When individuals receive certain social feedback, they will experience gain and loss of face. For example, when somebody is praised in public, he will feel that he has a face, while when somebody is criticized in public, he will feel that he has no face or lose face, which may lead to face pressure—the perception of individuals under face threat [28], that is, when people feel the risk and possibility of losing face, there is a perceived face threat.

Social situational factors are one of the crucial factors affecting face perception, in which social feedback or evaluation plays a key role in individuals’ perceived face threat [28]. Employees’ status in the workplace implies the evaluation of individual workability, which can significantly affect employees’ perceived face threat [28]. The fundamental purpose of the boss’s abusive behavior is to convey relevant information to the subordinates and correct the work deviation, and the subordinates’ poor work performance is an important reason for the abusive treatment [21]. Therefore, abusive supervision can be regarded as a critical social evaluation in the workplace, which can convey information about subordinates’ abilities and performance, and thus affect individuals’ perceived face threat. To be specific, public criticism and ridicule from the boss can easily make employees feel helpless and frustrated and hurt their self-esteem [21]. Employees also perceive their lack of ability and poor performance from the abusive behavior of their superiors; they perceive that they may lose the opportunity for a salary increase, promotion, or even their current status in the organization, which will lead to the loss of individual face and “lose face” in front of colleagues. These negative feelings will aggravate subordinates’ concerns about their status and image and make employees feel increasing potential risks and the possibility of “losing face”, resulting in a more significant threat to perceived face threat [28]. The following hypothesis was proposed accordingly:

**H1.**  *Abusive supervision is positively related to perceived face threat*.

### 2.2. Perceived Face Threat and Feedback Seeking

Feedback seeking is based on positive psychology, which points out that employees can actively seek feedback from superiors or colleagues to obtain valuable information for themselves, so as to promote the development of individuals and organizations. Furthermore, research has confirmed that employees who often actively seek feedback not only have a high degree of identification with the organization, can quickly integrate into the organization, and show good task performance and innovation performance at work, but also can better establish a high-quality relationship with their superiors and show lower turnover intentions [29]. Therefore, feedback-seeking behavior in an organization has a positive impact on both individual and organizational development.

According to the face theory, when an individual experiences the pressure of face loss, there will be a strong demand motivation to maintain or increase face, which will stimulate the individual to adopt a series of behavioral strategies, such as improving the ability, self-defense, etc., and expect to gain positive evaluation from others to win face [28]. On the one hand, gaining more face makes people feel the joy and pride of being respected; on the other hand, the “size” of face means a person’s social status [28]. According to the COR theory, resources are “precious objects with individual characteristics, conditions and energy that make individuals feel valuable.” In the interaction process between individuals and their social environment, individual behaviors under pressure are driven by the motivation to conserve and acquire resources [30]. Therefore, for individuals in an organization, face is a vital resource related to their value and status. According to the principle of primacy of resource loss, the psychological damage caused by resource loss to individuals is greater than the psychological help generated by resource acquisition. Therefore, individuals will first take countermeasures to avoid further resource loss in the face of resource loss—they are more likely to reduce their interaction with leaders to avoid further resource loss [12]. It can be seen that the motivation to maintain face resources is stronger than the motivation to obtain more face resources. Through evaluating the risk and possibility of losing face, subordinates will choose to avoid further communication with their superiors to reduce the further loss of face resources. The following hypothesis was proposed accordingly:

**H2.**  *Perceived face threat is negatively related to feedback seeking*.

**H3.**  *Perceived face threat plays a mediating role between abusive supervision and feedback seeking.*

### 2.3. Moderating Effect of Self-Affirmation

Self-affirmation refers to maintaining overall good and socially appropriate self-worth—self-integrity by thinking about other important self-worth unrelated to the threat domain so that people can see themselves from a broader perspective [31]. Alternatively, because important self-worth is anchored, information that threatens the self loses its threatening power, because people no longer focus on the threat of the information but on the value of the information itself. Therefore, threatening information can be processed and accepted in a more open, fair and objective way [32,33], which protects the self and does not lose the opportunity to learn knowledge from failure and correct wrong attitudes or behaviors [34,35].

However, self-affirmation cannot reduce the threat immunity of individuals [36], which means that the appearance of the self-affirmation effect is conditional. When the self-worth in the same field as the threat information is affirmed, the “same-domain effect”—the reverse effect of self-affirmation will appear, which may enhance the individual’s confidence and grasp of events, and thus more contradictory to the threat information [22,37]. As face is a social psychological construction rooted in East Asian culture and an important factor affecting the psychology and behavior of East Asians, especially Chinese people [35], the value implied among people is an important value field for Chinese people. In this regard, the famous Chinese writer and thinker Lu Xun also pointed out: “Face is the spiritual program of the Chinese people” [38]. Mr. Lin Yutang, a famous scholar, once said that “face, fate and favor are called the three goddesses who have ruled the Chinese nation since ancient times” [39]. Even in modern society, the important role of face for the Chinese people has not changed in nature [40]. In the workplace, resources and information controlled by leaders (performance appraisal, promotion, etc.) are very important to employees, so how leaders treat employees largely represents whether the employees contribute to the organization, whether they are valuable, and whether they are recognized [41]. Therefore, when employees suffer abusive supervision from superiors which further leads to the face threat, those employees with high self-affirmation who regard face as an important self-worth anchor area will more strongly perceive the abusive supervision as a threat to their face because of the reverse self-affirmation effect. The following hypothesis was proposed accordingly:

**H4.**  *Self-affirmation positively moderates the relationship between abusive supervision and perceived face threat.*

### 2.4. Moderating Effect of Self-Handicapping

Self-handicapping was first studied by Berglas and Jones (1978), who defined it as “any action or choice taken by an individual in a performance situation to avoid or reduce the negative impact brought by a poor performance that can increase the opportunity to externalize the cause of failure” [42]. Self-handicapping strategies can be divided into two categories: one is action self-handicapping, which refers to the behavioral strategies that individuals adopt in advance to make favorable attribution, such as drinking too much and reducing the time for practice. The other is self-reported handicapping, which refers to individuals claiming that some factors may affect their performance before undertaking a task, such as tension, anxiety, physical discomfort, etc. [43]. Studies have shown that individuals mainly adopt self-handicapping to protect their self-worth and divert people’s attention from their ability [44]. In addition, subjects’ self-handicapping tendency is significantly higher in public than in private [44]. Thus, self-handicappers are more concerned with their image in the eyes of others than how they actually perform.

Richards et al., (2002) found in their research that the presence or absence of others and the individual’s perception of the importance of performance results impact whether an individual uses self-handicapping strategies. The former is that the presence of others increases the motivation of self-presentation, thus arousing individuals’ desire to protect and enhance their self-worth. Therefore, they will try to use certain strategies to influence others’ perceptions of themselves and leave a good public image. The latter is because if the performance situation has nothing to do with self-concept, it is unlikely to provoke self-handicapping behavior [45]. However, when subordinates are subjected to abusive supervision by superiors, the perceived face threat will be aroused. Because on the one hand, the face represents social dignity or public image recognized by others in East Asian culture [35]; on the other hand, the expression form of abusive supervision is open. Therefore, when employees’ faces are threatened by abusive supervision, they may adopt self-handicapping strategies to protect their values. In particular, high self-handicappers prefer self-protection [25,26], which makes them more likely to reduce active efforts to achieve self-protection strategies after perceiving that their face representing their value is threatened. The following hypothesis was proposed accordingly:

**H5.**  *Self-handicapping moderates the relationship between subordinates’ perceived face threat and feedback seeking. That is, employees with high self-handicapping strengthen the negative relationship, while employees with low self-handicapping weaken the negative relationship.*

**Figure 1:**
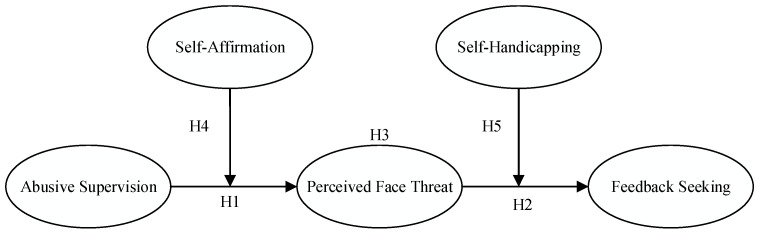
Conceptual Model.

In summary, the conceptual model of this study is shown in Figure 1.

## 3. Methods

### 3.1. Sample Collection and Sample Characteristics

The necessity of focusing on the sensitivity of abusive supervision and employment discrimination topics has been taken into account during the distribution and collection of the questionnaires. Moreover, we have selected the multi-source multi-period method to avoid deviations from the ordinary method. The process of obtaining respondents is as follows: (1) We randomly contacted manufacturing entrepreneurs through multiple entrepreneur clubs and MBA workshops and then conducted preliminary communication and contact to describe the intention of this program and pick out the enterprises willing to participate in this study. (2) The involved enterprises were distributed in many provinces and municipalities, such as Beijing, Jiangsu, Zhejiang, Gansu, Henan, Shanxi and Guangdong, thus ensuring the extensive geographical coverage of this study. (3) Most of these enterprises were manufacturing enterprises. Therefore, their employees and employees’ direct supervisors were chosen as respondents to the questionnaires. Because on the one hand, supervisors of enterprises in the manufacturing industry are more likely to be abusive to subordinates, and assembly line workers are considered to be one of the groups that suffer the most abusive behaviors from their leaders [46]; on the other hand, manufacturing enterprises focus on production efficiency, encouraging all activities for staff improvement, thereby providing a suitable work environment for feedback-seeking behaviors.

In order to ensure the process of sample collection and avoid deviation from the common method, we have collected survey data matched between employees and their direct supervisors in three periods. The process is specified as follows:

(1) Considering the sensitivity of abusive supervision topics and COVID-19 pandemic prevention and control in China, all questionnaires were distributed online. In order to ensure the match of survey data between supervisors and subordinates, the name list of surveyed employees and their direct supervisors was provided by each enterprise involved in this study, and the questionnaires were numbered according to the name list. Before each survey, we would provide each superior and his or her subordinates with a separate and unique online link containing a questionnaire. After login, the respondents could answer the questionnaire and submit it online. Meanwhile, they must promise to keep the survey results strictly confidential.

(2) After eliminating invalid questionnaires with wrong or missing information, too many single options or invalid matches, we have collected the following data in three periods: The first period was from 15 June to 20 June 2022. We collected the demographic variables of respondents, including gender, educational background, job title and working years of leaders, and gender, educational background, job title and current working years of employees, the moderating variable (self-affirmation, self-handicapping), and independent variable (abusive supervision). Considering that one leader corresponds to multiple subordinates, we distributed 676 questionnaires to subordinates and 95 questionnaires to leaders. All the survey data collected in the first period were matched according to the superior-subordinate relationship, thereby obtaining 454 valid questionnaires. We distributed the questionnaires for the second period to the successfully matched superiors and subordinates in the first period.

The second period was from 15 July to 20 July 2022. We collected the data of the mediating variable (perceived face threat). All the questionnaires collected in these two periods were matched according to the relationship between superiors and subordinates. Thus, we collected 382 valid questionnaires and distributed the questionnaires for the third period to the successfully matched superiors and subordinates in the first and second matches.

The third period was from 15 August 2022 to 20 August 2022. We collected the data of the dependent variable data (feedback seeking). The data successfully matched in the first and second periods were further matched with the data collected in the third period and invalid questionnaires were deleted. Finally, we obtained 318 valid questionnaires. See Table 1 for the demographic characteristics of the samples.

### 3.2. Variable Measurement and Test

All scales involved in this study have been published and proven to be effective mature scales in Chinese organizational situation studies. All scales were scored using a 5-point Likert scale. In the measurement of abusive supervision and feedback seeking, employees were asked to select the items that truly reflected their daily interactions with their superiors: 1 was “never happens”, 2 was “rarely happens”, 3 was “occasionally happens”, 4 was “sometimes happens”, and 5 was “often happens”. In the measurement of self-affirmation, self-handicapping and perceived face threat, employees are asked to select the items that can represent their real working status: 1 represents “very inconsistent”, 2 represents “not very consistent”, 3 represents “difficult to judge”, 4 represents “fairly consistent”, and 5 represents “very consistent”.

Abusive supervision: We applied the 10-item scale developed by Aryee et al., (2008) [47], such as “My leader laughed at me” and “My leader was rude to me”. The α coefficient of this scale in this study is 0.94.

Perceived face threat: We applied the 11-item scale developed by Zhao et al., (2020) [28], such as “I think my working ability will be questioned by others” and “Avoiding such interaction with leaders will make others suspect that I lack the necessary knowledge or ability”. The α coefficient of this scale in this study is 0.95.

Feedback seeking: We applied the 5-item scale developed by VandeWalle et al., (2000) [48], such as “I ask my direct supervisor for feedback on my overall performance” and “I ask my direct supervisor for feedback on the technical aspects of my work”. The α coefficient of this scale is 0.94.

Self-affirmation: We applied the 9-item scale developed by Li Hong et al., (2002) [49], such as “Generally speaking, I am quite happy with everything”, “I can concentrate on everything”, and “I am very satisfied with the way I do things”. The α coefficient of this scale is 0.81.

Self-handicapping: We applied the 14-item scale developed by Rhodewalt (1990) [50], such as “When I do something wrong, my first reaction is to blame the environment” and “I often leave things to the last minute”. The α coefficient of this scale is 0.85.

Control variables: Previous studies have shown that employees’ gender, educational background, and working years have a certain influence on their feedback-seeking behavior [14,51]. Therefore, this paper selects the gender, educational background and working years of employees as control variables and adopts the continuous coding method for classification. The gender code is 0 for males and 1 for females. The education code we set college and below college for 1; undergraduate is 2; master or above is 3; working years is a continuous variable.

## 4. Data Analysis and Research Results

### 4.1. Confirmatory Factor Analysis and Common Method Variance Analysis

Following the standard empirical testing process, Mplus8.3 software was used to conduct confirmatory factor analysis on the variables involved in the theoretical research model (abusive supervision, perceived face threat, feedback seeking, self-affirmation and self-handicapping). Since there were many items in the measurement of self-handicapping and perceived face threat, the items were packaged according to the suggestions of Wu and Wen (2011) [52]. The perceived face threat variables were packaged into four sub-dimensions according to the original sub-dimensions of the scale (four sub-dimensions of the perceived face threat: perceived ability face threat, perceived relational face threat, perceived moral face threat, and perceived autonomous face threat). The self-handicapping variables were packaged into 4-items by the equilibrium method. Table 2 shows that compared with the alternative model, the five factors model has a better fitting (χ^2^/df = 2.69, CFI = 0.90, TLI = 0.89, RMSEA = 0.07, SRMR = 0.07).

In addition, although this paper adopts the multi-time point pairing method to collect data to avoid the problem of common method bias, we still consider it necessary to summarize the validity of the test data from the test results. Harman’s single potential factor method was used in this study to test the common method bias. The results are shown in Table 2, and all the goods-of-fit indexes showed that the one-factor model was poorly fitted (χ^2^/df = 11.23, CFI = 0.39, TLI = 0.35, RMSEA = 0.18, SRMR = 0.19). However, the chi-square value differences also indicate that the fit of the five factors model is significantly better than that of the one-factor model (∆χ^2^ =3981.44, ∆df = 8, *p* < 0.01), further indicating that the common method bias is not a problem in this data.

### 4.2. Descriptive Statistics and Correlation Analysis

Table 3 shows each variable’s mean value, standard deviation and Pearson correlation coefficient. The correlation analysis results were as follows: (1) Abusive supervision was significantly positively correlated with perceived face threat (r = 0.20, *p* < 0.01), and abusive supervision was significantly negatively correlated with feedback seeking (r = −0.15, *p* < 0.01); (2) Perceived face threat was negatively correlated with feedback seeking (r = −0.19, *p* < 0.01); The results of correlation test of each variable lay a foundation for the follow-up test.

### 4.3. Hypothesis Test

#### 4.3.1. Mediating Effect Test

**Figure 2:**
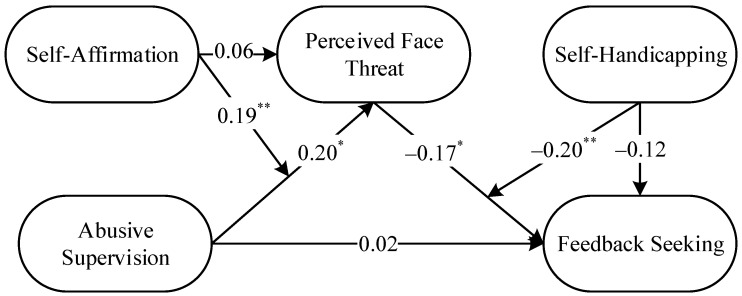
Results of the path analysis. Note. The path coefficients in the figures are not standardized; the path coefficient of standardizing the influence of demographic variables on feedback-seeking is not shown in the figure; * = *p* < 0.05, ** = *p* < 0.01; N = 318.

This study used a path analysis model using Mplus 8.3 software for subsequent hypothesis testing. A full model with abusive supervision as the independent variable, perceived face threat as the mediating variable, feedback seeking as the dependent variable and self-affirmation and self-handicapping as the moderating variables (shown in Figure 1) was constructed, and a path analysis model was calculated. The results of the path analysis are shown in Figure 2.

First, the calculations using Mplus 8.3 software revealed a positive effect of abusive supervision on perceived face threat (B = 0.20, *p* < 0.05) and a significant negative effect of perceived face threat on feedback-seeking (B = −0.17, *p* < 0.05); therefore, H1 and H2 were supported by the results. Notably, the direct effect of abusive supervision on feedback-seeking was not significant (B = 0.02, n.s.), thus tentatively verifying that perceived face threat played a complete mediating role between abusive supervision and feedback-seeking. Second, this study further validated the results of the path analysis using the bootstrapping test, the results of which are shown in Table 4. The direct effect of abusive supervision on feedback-seeking was not significant at the 95% confidence interval (Effect = 0.02, SE = 0.07, CI = [−0.12, 0.16]). However, the indirect effect of abusive supervision on feedback-seeking was significant (Indirect Effect = −0.04, SE = 0.02, CI = [−0.10, −0.003]). The results of the bootstrapping test further validated the complete mediating role of perceived face threat in the relationship between abusive supervision and feedback seeking. Therefore, H3 was also supported by the results.

#### 4.3.2. Moderating Effect Test

This section also used path analysis to test the moderating effects of self-affirmation (Hypothesis 4) and self-handicapping (Hypothesis 5). The results are shown in Figure 2: (1) the direct effect of self-affirmation on perceived face threat was not significant (B = 0.06, n.s.), while the interaction term between self-affirmation and abusive supervision had a significant positive effect on perceived face threat (B = 0.19, *p* < 0.01); (2) similarly, the direct effect of self-handicapping on feedback-seeking was not significant (B = −0.12, n.s.), while the interaction term between self-handicapping and perceived face threat had a significant negative effect on feedback-seeking (B = −0.20, *p* < 0.01). That is, there was preliminary evidence of a significant moderating effect of self-affirmation between abusive supervision and perceived face threat and a significant moderating effect of self-handicapping between perceived face threat and feedback seeking.

**Figure 3:**
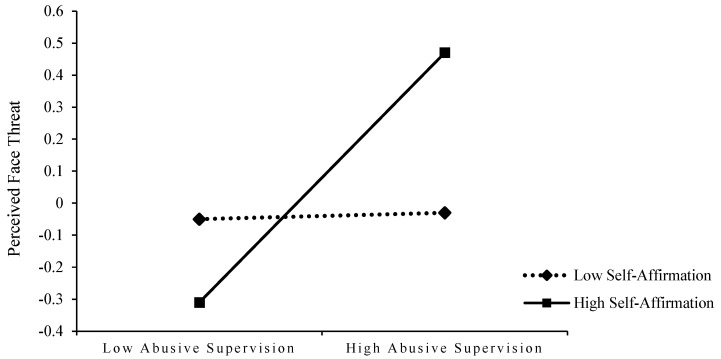
The interactive effects of abusive supervision and self-affirmation on perceived face threat.

**Figure 4:**
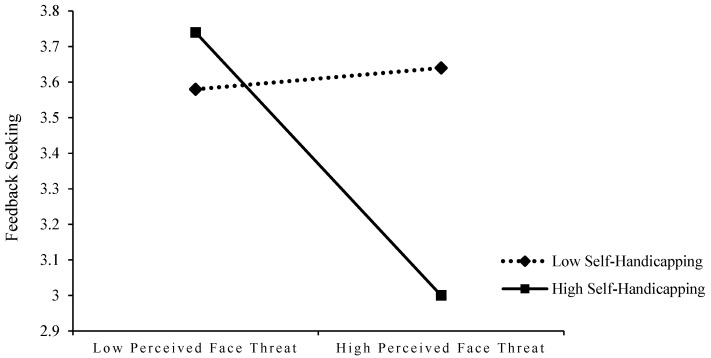
The interactive effects of perceived face threat and self-handicapping on feedback seeking.

In addition, to better explain the moderating effects of self-affirmation and self-handicapping, the sample was divided into high and low self-affirmation groups (mean self-affirmation ± one standard deviation) and high and low self-handicapping groups (mean self-handicapping ± one standard deviation), and the moderating effects of self-affirmation and self-handicapping were plotted using simple slope analysis. As shown in Figure 3 and Figure 4: (1) for subjects with low self-affirmation, the positive effect of abusive supervision on perceived face threat was not significant (B = 0.01, n.s.),whereas, for subjects with high self-affirmation, this relationship was positively significant (B = 0.40, *p* < 0.01), i.e., indicating that self-affirmation enhanced the positive effect of abusive supervision on perceived face threat and that this positive effect was only for employees with higher levels of self-affirmation; (2) similarly, perceived face threat was significantly negatively related to feedback seeking for subjects with higher levels of self-handicapping (B = −0.37, *p* < 0.01); whereas for subjects with lower levels of self-handicapping, this negative relationship was not significant (B = 0.02, n.s.). That is, it suggests that self-handicapping enhances the negative effect of perceived face threat on feedback-seeking and that this negative effect is only significant for employees with higher levels of self-handicapping. Therefore, both H4 and H5 were supported by the results.

## 5. Discussion and Application

### 5.1. Theoretical Implications

Based on face theory and resource conservation theory, this study explores the mediating role of perceived face threat in the relationship between abusive supervision and feedback seeking. Further, it analyzes the boundary-regulating effects of self-affirmation and self-handicapping in this mechanism. The results showed that abusive supervision had a negative impact on employees’ feedback-seeking behavior by positively affecting perceived face threat, self-affirmation positively moderated the relationship between abusive supervision and perceived face threat, and self-handicapping enhanced the negative impact of perceived face threat on feedback-seeking.

The theoretical significance of this study is as follows. First, this study expands the theoretical framework for explaining the effect of abusive supervision on feedback-seeking behavior. Although some studies have pointed out that abusive supervision will reduce employees’ feedback-seeking behavior, the mechanism of action is rarely clarified. Only Shen et al., (2020) pointed out that abusive supervision will reduce employees’ feedback-seeking behavior by affecting their organizational self-esteem [17]. This study focuses on organizational management in the Chinese cultural environment where the concept of face is more important and introduces the perceived threat of face into the explanatory framework of feedback seeking in abusive supervision, thus enriching the existing theoretical research. In addition, studies have shown that because individuals are motivated to gain face, a series of positive behaviors conducive to “face earning” will occur after being subjected to abusive supervision by superiors. However, based on the theory of resource conservation, this paper points out through research that face selection strategies related to face loss will focus on how to maintain, save or avoid face loss, thus resulting in withdrawal behaviors such as not taking actions—reducing feedback seeking. Meanwhile, this paper reveals how abusive supervision negatively impacts employees’ positive behaviors by affecting their faces and enriches the existing research.

Second, this study has further enriched one of the essential research propositions in this field pointed out by Tepper, the author of abusive supervision, namely, the explanatory framework for the difference of abusive perception [21]. As abusive supervision is a kind of negative pressure stimulus for employees and threatens their self-worth perception, it will trigger the motivation to protect their self-worth. In this paper, two important individual characteristic variables, self-affirmation and self-handicapping, are introduced to reveal the important moderating effects of abusive supervision on employees’ feedback-seeking behavior. In particular, it points out that because face is an important area in the construction of Chinese people’s self-worth, self-affirmation has the reverse effect of the “same-domain effect” in the impact of abusive supervision on employees’ perceived face threat, which not only does not cushion the negative impact of abusive supervision on employees, but strengthens its positive impact on victims’ perceived face threat. However, through the potentially defensive attribution pattern, individuals with high self-handicapping become more accustomed to external attribution after being abused by superiors, attribute unfair treatment of superiors to environmental and external factors more often and make negative avoidance behaviors to protect self-worth after perceiving face threats, thus reducing feedback-seeking behaviors. This reveals that the difference between employees’ self-affirmation and self-handicapping levels may be an important factor affecting employees’ perceived level of abusive supervision and an important boundary moderating variable in the process of the effect of abusive supervision on employees’ feedback-seeking behavior.

Third, it enriches the research framework of self-affirmation, the “same-domain effect” reaction mechanism. Numerous studies have pointed out that self-affirmation can help individuals maintain their self-integrity when confronted with threats by affirming their self-worth in fields unrelated to threats; that is, individuals think that they are generally good [32]. Only a few pieces of literature indicate that the emergence of this positive effect is conditional, and the boundary of self-affirmation is affected by the “in-domain effect”, but the research in the field of value anchoring is insufficient. This study points out that face is an important value domain of Chinese people in Chinese organizations that attach importance to face culture. When face is threatened, it may lead to negative behaviors of individuals with a high level of self-affirmation, which enriches the research in the field of value anchoring of self-affirmation.

### 5.2. Practical Significance

In the fierce market competition, the influence and control of leaders on enterprises play a crucial role in the survival and development of enterprises [53,54]. However, more and more studies have pointed out that a large number of destructive leadership behaviors exist in organizations, such as abusive supervision [27,55], which brings numerous negative impacts to both enterprises and employees [56,57,58,59,60]. This paper further confirms this point through empirical research that abusive supervision will make employees feel face threat and thus reduce employees’ upward feedback-seeking behavior, which will have a negative impact on the sustainable development of both organizations and employees.

Therefore, according to the results of this study, first of all, we call on organizations to strengthen the moral education of managers and strengthen the supervision and management of their workplace behavior, so as to reduce or eliminate abusive supervision. This is because although a small number of studies have supported the positive effects of abusive supervision, a large number of scholars have pointed out that abusive supervision has caused a series of negative physical and mental impacts on employees, and even leads to negative behaviors against their superiors and organizations, thus bringing huge economic losses to enterprises every year [54]. In addition, under the United Nations Sustainable Development Goals initiative, organizations have a responsibility and obligation to promote a safe and secure work environment and take care of the physical and mental well-being of employees in the organization and help them to work decently [61,62,63]. Only then will organizations be better able to achieve sustained, inclusive, and sustainable economic growth (Available online: https://www.un.org/sustainabledevelopment/zh/sustainable-development-goals/ (accessed on 27 May 2023)).

In addition, this study suggests that leaders criticize employees for specific deficiencies in their work and possible causes, rather than disparaging their overall level or accusing their personality flaws. Especially in China, where the face culture is influential, even if leaders criticize subordinates for the pro-organizational motivation of improving organizational performance, public criticism and criticism should be avoided. Because this will stimulate employees with high self-affirmation traits to further aggravate the perceived face threat and ultimately reduce subordinates’ proactive feedback-seeking behavior, which is not conducive to employee improvement and growth. Enterprise managers should pay attention to employees’ demand for face and induce them to implement a series of behaviors expected by the organization by stimulating their motivation to “maintaining face” and “increasing face”.

Third, the use of abusive supervision by leaders should vary from person to person. Employees with high self-handicapping tend to be more prone to external attribution and are more sensitive to external evaluation and the environment. For employees with high self-handicapping, enterprises should increase staff mental health training, pay attention to their self-esteem and affirm their value. In this way, employees suffering from abusive leadership will be avoided to produce greater psychological pressure and further stimulate the employees’ self-defense psychology. Especially at work, managers should take the initiative to strengthen the identification of employees’ characteristics and give corresponding guidance and relief to employees with high self-handicapping, so that employees can view criticism rationally, comprehensively analyze their advantages and disadvantages, face the criticism with a learning attitude, better adjust the pressure, and then find mistakes and try to improve work performance.

### 5.3. Limitations and Prospects

Although this research has certain theoretical and practical value, there are still some limitations.

First, this study reveals that overall perceived face threat completely mediates the relationship between abusive supervision and feedback seeking. However, the perceived face threat contains four dimensions: the perceived ability face threat, the perceived relationship face threat, the perceived moral face threat, and the perceived autonomous face threat. In the future, the dimensions of the perceived face threat can be refined to verify the role of different dimensions of face threat in the relationship between abusive supervision and subordinates’ behavior, and further improve the abusive supervision—face pressure—individual behavior research.

Second, from the perspective of the situations and individual characteristics that trigger the perceived face threat, this paper mainly focuses on the moderating effects of self-affirmation and self-handicapping on the effect of abusive supervision. However, studies on self-defense induced by stress and threat indicate that there are more complex and diverse boundary conditions in this mechanism. Therefore, we believe that in the future, we can further improve the boundary conditions of the influence of abusive supervision on subordinates’ perceived face threat from the influence of other individual characteristics of subordinates on the relationship between abusive supervision and perceived face threat.

Third, the sample data of this study are all from local enterprises in different regions and industries in China, so the research conclusions have certain limitations. In particular, considering that face concerns are not only a unique phenomenon in China and East Asia, more cross-regional countries should be carried out in the follow-up research on the threat of abusive supervision to employees’ perceived face, which will further test the conclusions of this study and provide more management countermeasures for different cultural characteristics.

## Figures and Tables

**Table 1:** Sample demographic characteristics distribution.

Characteristic	Form	N	%
Gender of Leadership	Male	45	81.8
	Female	10	18.2
Education of Leadership	College and below	24	43.6
	Undergraduate	26	47.3
	Master or above	5	9.1
Professional field of Leadership	Technology	17	30.9
	Marketing	4	7.3
	Management	5	9.1
	Finance	3	5.5
	Production	23	41.8
	Other	3	5.5
Gender of Employee	Male	187	58.8
	Female	131	41.2
Education of Employee	College and below	203	63.8
	Undergraduate	111	34.9
	Master or above	4	1.3
Professional field of Employee	Technology	82	25.8
	Marketing	9	2.8
	Management	26	8.2
	Finance	10	3.1
	Production	169	53.1
	Other	22	6.9

**Table 2:** Results of confirmatory factor analysis.

Model	χ^2^	df	χ^2^/df	CFI	TLI	RMSEA	SRMR
Five-factor model: AS,PFT,FS,SA,SH	1228.21	456	2.69	0.90	0.89	0.07	0.07
Four-factor model1:AS + SH,PFT,FS,SA	1795.44	459	3.91	0.83	0.82	0.10	0.09
Four-factormodel2:AS + PFT,FS,SA,SH	2159.24	459	4.70	0.78	0.76	0.11	0.11
Four-factormodel3:AS + SA,PFT,FS,SH	2278.92	459	4.97	0.77	0.75	0.11	0.13
Four-factor model4:AS + FS,PFT,SA,SH	2676.40	459	5.83	0.72	0.69	0.12	0.13
One-factor model: AS + PFT + FS + SA + SH	5209.65	464	11.23	0.39	0.35	0.18	0.19

Note. AS = Abusive Supervision, PFT = Perceived Face Threat, FS = Feedback Seeking, SA = Self-Affirmation, SH = Self-Handicapping, + Indicates Fusion.

**Table 3:** Descriptive statistics and correlation coefficients of each variable.

Variables	M	SD	1	2	3	4	5	6	7	8
1. Gender	0.45	0.56	-							
2. Education	1.37	0.51	0.12 *	-						
3. Working Years	11.20	10.42	−0.13 *	−0.21 **	-					
4. AS	1.84	0.74	−0.02	−0.02	0.06	-				
5. PFT	2.31	0.91	0.02	−0.16 **	0.19 **	0.20 **	-			
6. FS	3.33	0.98	−0.06	−0.003	−0.06	−0.15 **	−0.19 **	-		
7. SA	3.80	0.55	−0.04	−0.05	0.04	−0.13 **	0.01	0.07	-	
8. SH	2.75	0.63	−0.01	0.11	0.02	0.27 **	0.08	−0.15 **	0.03	-

Note. AS = Abusive Supervision, PFT = Perceived Face Threat, FS = Feedback Seeking, SA = Self-Affirmation, SH = Self-Handicapping; * = *p* < 0.05, ** = *p* < 0.01.

**Table 4:** Results of the bootstrapping analysis.

Path	Effect	SE	95%CI	
			95%LL	95%LL
Direct Effect				
Abusive Supervision → Feedback Seeking	0.02	0.07	−0.12	0.16
Mediating Effect				
Abusive Supervision → Perceived Face Threat → Feedback Seeking	−0.04	0.02	−0.10	−0.003

Note. Bootstrapping = 5000.

## Data Availability

The data presented in this study are available on request from the corresponding and third author.
